# Thrombus Formation in Left Atrium on Dabigatran Therapy

**DOI:** 10.1155/2015/518982

**Published:** 2015-02-22

**Authors:** Priyank Shah, Priyam Mithawala, Donna Konlian, Aderemi Soyombo, Mahesh Bikkina

**Affiliations:** ^1^St. Joseph's Regional Medical Center, 703 Main Street, Paterson, NJ 07503, USA; ^2^Ohio State University, Columbus, OH 43210, USA

## Abstract

Dabigatran is a direct thrombin inhibitor, approved in the United States for stroke prevention in nonvalvular atrial fibrillation and prevention and treatment of thromboembolism. It has been also used in patients with documented left atrial thrombus, where treatment with dabigatran effectively led to thrombus resolution. We present a rare case of left atrial thrombus formation in a patient with chronic atrial fibrillation being treated with dabigatran 150 mg twice a day. The patient presented with multiple embolic strokes. There are only three such cases reported in the literature till date, all of whom had thrombus in the left atrium. The possible mechanisms of dabigatran failure include compensatory increase in upstream coagulation factors due to single level downstream blockade of thrombin, lack of inhibition of all available thrombin, and lack of monitoring measures that can be implemented in common clinical laboratories that lead to failure to assess adherence, which in turn can lead to dabigatran failure.

## 1. Introduction 

Dabigatran is a direct thrombin inhibitor. It binds to thrombin in competitive and reversible manner. Binding of dabigatran to thrombin prevents catalytic conversion of fibrinogen to fibrin by thrombin. It also inhibits thrombin-mediated activation of platelets and amplification of coagulation cascade [1, 2]. The half-life dabigatran is 12–17 hours in healthy subjects without renal dysfunction. It follows linear pharmacokinetics and pharmacodynamics after oral administration two times a day. Dabigatran was approved in the United States in 2010 for nonvalvular atrial fibrillation based on the results of the RE-LY trial [3]. Although dabigatran 150 mg twice daily reduced the primary outcome of stroke/systemic embolism by 34% compared to warfarin in RE-LY trial [3], failure of this therapy can occur in occasional cases. We report one such rare case of left atrial thrombus formation in a patient with chronic atrial fibrillation on dabigatran therapy.

## 2. Case Report 

A 60-year-old male with past medical history of hypertension, coronary artery disease, chronic atrial fibrillation, and stroke (in 2011) presented to the hospital with complaints of slurred speech, dizziness, and gait imbalance in October 2014. He denied any other complaints. His blood pressure on admission was 180/102 mm Hg and heart rate was 77/minute. Physical examination was significant for left facial droop and irregularly irregular heart sounds. The rest of the physical examination was normal. Electrocardiogram showed atrial fibrillation with ventricular rate of 80 beats per minute. A noncontrast CT scan of the brain showed new lacunar infarct in right corona radiata and old right frontal lobe infarct. Subsequent MRI of the brain showed acute infarcts in right insular cortex, subinsular region, and right corona radiata along with an old right frontal lobe infarct. His laboratory tests were within normal limits. The patient was continued on dabigatran 150 mg twice daily, which he was taking at home. His other home medications included aspirin 81 mg, metoprolol succinate 100 mg, diltiazem sustained release 120 mg, and atorvastatin 40 mg daily. The patient had a regular follow-up with his cardiologist and was complianing with all his medications. He had a transesophageal echocardiogram done in August 2011, which did not reveal any smoke or clot in left atrium or left atrial appendage. He subsequently underwent electrical cardioversion for atrial fibrillation at that time but reverted back to atrial fibrillation shortly thereafter. He has been on dabigatran since then.

**Figure 1:**
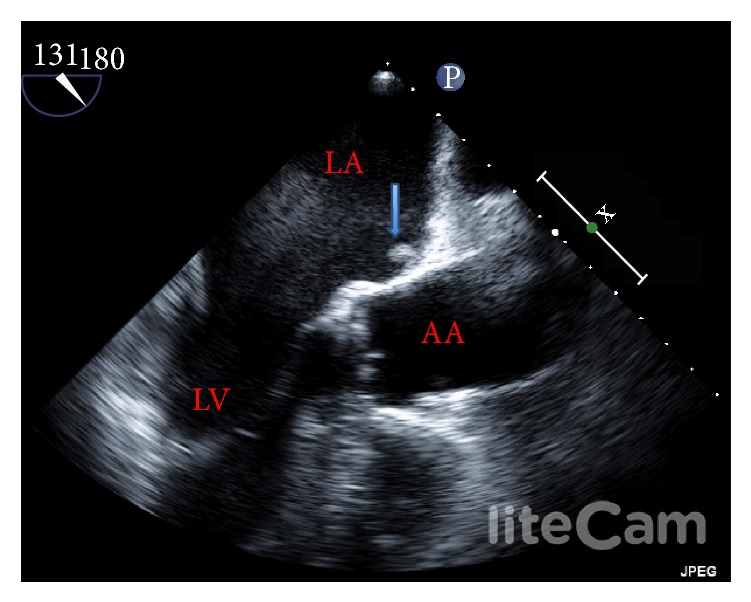
Transesophageal echocardiogram showing a thrombus in the left atrium (arrow). LA: left atrium, LV: left ventricle, and AA: ascending aorta.

A transesophageal echocardiogram was done on this admission to evaluate a likely embolic source of acute stroke. Surprisingly, it revealed significant smoke and a mobile thrombus in the left atrium measuring about 0.6 cm × 0.3 cm (Figure 1, Video 1 (see Video 1 in Supplementary Material available online at http://dx.doi.org/10.1155/2015/518982)). The left atrial appendage was free of thrombus. The patient was switched to warfarin anticoagulation for presumed dabigatran failure. The rest of his hospital course was unremarkable and he was discharged to acute rehabilitation after 3 days on warfarin anticoagulation along with low molecular weight heparin bridging.

## 3. Discussion

Since its approval in 2010, dabigatran has been an effective alternative to warfarin in patients with nonvalvular atrial fibrillation. It has also been used in patients with atrial fibrillation who had documented left atrial thrombus, proving its role in resolution of thrombus [4, 5]. However, left atrial thrombus formation on dabigatran therapy as in our case has been reported in 3 other cases in the literature till date [6, 7]. Sharma et al. [6] recently reported two cases of left atrial thrombus formation on dabigatran therapy for chronic atrial fibrillation. One of the patients had lung cancer which could have contributed to additional risk of thrombosis, while the other patient was similar to our case. Our patient was very compliant with his medications and was not taking any medications, which could have decreased the bioavailability or effectiveness of dabigatran. Luis et al. [7] reported another case of left atrial thrombus on dabigatran therapy in a middle-aged female with rheumatic mitral stenosis and atrial fibrillation. This was however an off-label use of dabigatran because the patient had valvular atrial fibrillation, for which the drug is not approved. In all four cases (including ours), the interesting finding was formation of thrombus in the left atrium but not the left atrial appendage.

Although dabigatran 150 mg twice daily was superior to warfarin for prevention of stroke or systemic embolism in RE-LY trial, 159 out of 6015 patients in 110 mg twice daily arm and 111 out of 6076 patients in 150 mg twice daily arm had ischemic/unspecified stroke [3]. It is unknown how many of these patients had embolic stroke and how many had left atrial or appendage thrombus while on dabigatran therapy.

It is not entirely clear why our patient and other 3 cases reported in the literature had left atrial thrombus on dabigatran therapy. However, some of the proposed mechanisms are as follows. First, single level downstream inhibition of thrombin can lead to compensatory increase in upstream clotting factors, which can predispose to thrombus formation. Second, all the available thrombin might not be inhibited. Third, absence of routine monitoring to assess adherence as well as to ensure therapeutic anticoagulation level exists in vivo. Lack of adherence can definitely lead to failure but sometimes therapeutic levels are not achieved despite proper adherence either due to drug-drug interactions or inadequate absorption. Again, in which patients these mechanisms play a role and which patients are susceptible to dabigatran failure is not known at this time. The best strategy of anticoagulation in patients who develop thrombus on dabigatran therapy is also not known. However, a tried and tested therapy with warfarin should be used in such patients.

## Supplementary Material

The Supplementary Video shows transesophageal echocardiogram in the midesophageal long axis view. There is significant smoke in the left atrium and a mobile thrombus can be seen in the left atrium.
